# Impact of tumour *RAS/BRAF* status in a first-line study of panitumumab + FOLFIRI in patients with metastatic colorectal cancer

**DOI:** 10.1038/bjc.2016.343

**Published:** 2016-10-20

**Authors:** Meinolf Karthaus, Ralf-Dieter Hofheinz, Laurent Mineur, Henry Letocha, Richard Greil, Josef Thaler, Eva Fernebro, Kelly S Oliner, Michael Boedigheimer, Brian Twomey, Ying Zhang, Gaston Demonty, Claus-Henning Köhne

**Affiliations:** 1 Klinikum Neuperlach/Klinikum Harlaching, Oskar-Maria-Graf-Ring 51, D81737 Munich, Germany; 2 Universitätsmedizin Mannheim, Theodor-Kutzer Ufer 1-3, 68167 Mannheim, Germany; 3 Institut Sainte-Catherine GI and Liver Cancer Unit, 84 000 Avignon, France; 4 Oncology Clinic, Västmanland's Hospital, 721 89 Västerås, Sweden; 5 IIIrd Medical Department, Paracelsus Medical University Salzburg and CCCIT Salzburg Cancer Research Institute, Müllner Hauptstrasse 45, 5020 Salzburg, Austria; 6 Klinikum Wels-Grieskirchen, Grieskirchner Straße 42, A-4600 Wels, Austria; 7 Central Hospital, Strandvägen 8, 35185 Växjö, Sweden; 8 Formerly of Amgen Inc., 1 Amgen Center Dr MS 30E-2-C, Thousand Oaks, CA 91320, USA; 9 Amgen Inc., 1 Amgen Center Dr MS 30E-2-C, Thousand Oaks, CA 91320, USA; 10 Amgen GmbH, Dammstrasse 23, 6301 Zug, Switzerland; 11 Onkologie Klinikum Oldenburg, Rahel-Straus-Str. 10, 26133 Oldenburg, Germany

**Keywords:** panitumumab, metastatic colorectal cancer, *RAS*, *KRAS*, *NRAS*, *BRAF*, amphiregulin, response

## Abstract

**Background:**

To investigate tumour biomarker status and efficacy of first-line panitumumab+FOLFIRI for metastatic colorectal carcinoma (mCRC).

**Methods:**

154 patients received first-line panitumumab + FOLFIRI every 14 days. Primary end point was objective response rate (ORR). Data were analysed by tumour *RAS (KRAS/NRAS)* and *BRAF* status, and baseline amphiregulin (AREG) expression.

**Results:**

Objective responses occurred more frequently in *RAS* wild type (WT) (59%) *vs RAS* mutant (MT) (41%) mCRC and in *RAS* WT/*BRAF* WT (68%) *vs RAS* or *BRAF* MT (37%) disease. Median response duration was longer in *RAS* WT (13.0 months) *vs RAS* MT (5.8 months) (hazard ratio (HR): 0.16). Median progression-free survival was longer in *RAS* WT *vs* MT (11.2 *vs* 7.3 months; HR, 0.37) and was also longer in *RAS* WT/*BRAF* WT *vs RAS* or *BRAF* MT (13.2 *vs* 6.9 months; HR, 0.25). Incidence of adverse events was similar regardless of *RAS/BRAF* status, and no new safety signals were noted. Among patients with *RAS* WT tumours, ORR was 67% with high AREG expression and 38% with low AREG expression.

**Conclusions:**

First-line panitumumab+FOLFIRI was associated with favourable efficacy in patients with *RAS* WT and *RAS* WT/*BRAF* WT *vs* MT mCRC tumours and was well tolerated.

FOLFIRI (folinic acid, infusional 5-fluorouracil, and irinotecan) is a recommended first- and second-line chemotherapy backbone in the treatment of metastatic colorectal cancer (mCRC) (National Comprehensive Cancer Network, 2015). Following a recent review by the European Medicines Agency (EMA), both of the epidermal growth factor receptor (EGFR) inhibitors – panitumumab and cetuximab – are now approved in Europe for first-line use in combination with FOLFIRI or FOLFOX (folinic acid, infusional 5-fluorouracil, and oxaliplatin) in patients with *RAS* wild-type (WT) mCRC.

In the first-line setting, EGFR inhibitors were originally indicated in patients with *KRAS* WT mCRC in combination with either FOLFOX (panitumumab or cetuximab) or FOLFIRI (cetuximab). Subsequently, mutations were identified in *KRAS* and *NRAS* in 17% of patients with non-mutated *KRAS* exon 2 in the phase III PRIME trial of panitumumab + FOLFOX *vs* FOLFOX alone (Douillard *et al*, 2013). As a result, EGFR inhibitor use was refined to include only those patients with *RAS* WT disease. At the same time, studies also identified *BRAF* as an important negative prognostic – though not predictive – marker for survival in patients with mCRC, regardless of treatment (Phipps *et al*, 2012; Douillard *et al*, 2013; Peeters *et al*, 2013, 2014a, c). Tumour expression of the biomarker amphiregulin (AREG) has also been correlated with survival during anti-EGFR therapy (Jacobs *et al*, 2009; Loupakis *et al*, 2014).

The improved risk–benefit profile of EGFR inhibitor treatment in patients selected by *RAS* status was later verified in trials of first- and second-line FOLFOX and FOLFIRI in combination with panitumumab or cetuximab (Douillard *et al*, 2013; Heinemann *et al*, 2014a, 2014b; Venook *et al*, 2014; Bokemeyer *et al*, 2015; Van Cutsem *et al*, 2015). Importantly, patients with *RAS* mutant (MT) tumours showed no improvement in efficacy with the addition of EGFR inhibitor compared with chemotherapy alone. Indeed, there is some evidence that EGFR inhibitors combined with FOLFOX in such patients are detrimental compared with FOLFOX alone (Douillard *et al*, 2013; Bokemeyer *et al*, 2015), and EGFR inhibitors should therefore not be given to patients with *RAS* MT tumours.

Panitumumab plus FOLFIRI demonstrated superiority over FOLFIRI alone in a second-line phase II trial that demonstrated improved progression-free survival (PFS) in patients with *RAS* WT mCRC, although overall survival (OS) was not significantly different in this study (Peeters *et al*, 2014b). The only published study of first-line panitumumab plus FOLFIRI in mCRC is a single-arm trial that indicated the efficacy and predictable safety profile of the combination in *KRAS* WT patients (Köhne *et al*, 2012), although preliminary data have been presented from a phase II study in which panitumumab plus FOLFOX4 or FOLFIRI was evaluated in patients with *KRAS* WT colorectal cancer and liver-limited disease (Abad *et al*, 2014). Here, we present a retrospective analysis of this first-line trial of panitumumab plus FOLFIRI, reporting efficacy and safety data for first-line FOLFIRI plus panitumumab according to tumour *RAS/BRAF* status and AREG levels in patients with mCRC.

## Patients and methods

### Study design

This is a retrospective analysis of data from a phase II, single-arm study (NCT 00508404); full details of the study design have been presented previously (Köhne *et al*, 2012). In brief, first-line panitumumab (6 mg kg^−1^) + FOLFIRI were administered every 14 days until progression, unacceptable toxicity or withdrawal of consent. If FOLFIRI was withdrawn or suspended as a result of toxicity, panitumumab could be continued, and vice versa.

### Patients

Patients were adults with histologically or cytologically confirmed, radiologically measurable mCRC and an Eastern Cooperative Oncology Group (ECOG) performance status of 0–2. Patients could be enrolled only if all disease sites were evaluated within 28 days before enrolment, and tissue from the primary or metastatic site was available. Those who had received prior systemic therapy (including anti-EGFR therapy) for mCRC (except adjuvant fluoropyrimidine-based chemotherapy given ⩾ 6 months before enrolment) were excluded. Full inclusion and exclusion criteria have been reported previously (Köhne *et al*, 2012).

The study protocol was approved by the relevant independent ethics committees. The study was conducted in accordance with the regulations and guidelines of the International Conference on Harmonisation of Good Clinical Practice. All patients provided signed, informed consent before any study-related procedures were performed.

### End points and analyses

The primary end point was the objective response rate (ORR) assessed using modified Response Evaluation Criteria in Solid Tumors (mRECIST v1.0) (Therasse *et al*, 2000). Secondary end points included disease control rate (DCR), duration of response (DoR), depth of response (DpR; defined as the percentage of tumour shrinkage at nadir or progression), PFS, and time to progression (TTP). Resection rates were also evaluated, as was early tumour shrinkage (ETS), defined as the percentage of patients with ⩾30% or ⩾20% tumour shrinkage at week 8 (exploratory analysis). Safety was evaluated in terms of the incidence and severity of adverse events (AEs), using the National Cancer Institute Common Toxicity Criteria version 3.0.

Data were analysed descriptively by tumour *RAS/BRAF* status. Tumour specimens were assayed for mutations in *KRAS* exons 3–4, *NRAS* exons 2–4 and *BRAF* exon 15 by bidirectional Sanger sequencing. Mutations in *KRAS* exon 2 were analysed by CE-marked DxS kit.

Baseline tumour AREG levels were analysed in the *RAS* WT and MT populations. Total RNA was extracted from formalin-fixed paraffin-embedded tissue samples and AREG expression levels were analysed by qualified reverse transcription quantitative polymerase chain reaction (RT-qPCR) assays (see Supplementary Material for details). A cutoff point for AREG status was prespecified based on analysis of data from an earlier clinical trial (STEPP) (Lacouture *et al*, 2010). Cox proportional hazards (PH) models were used to evaluate AREG expression levels as a continuous covariate. Decision curves were used to estimate the PFS hazard ratio (HR) with increasing levels of baseline AREG expression. A Gaussian Process (GP) model was used to fit the PH model (Joensuu *et al*, 2012) using the GPstuff toolkit in MATLAB (Vanhatalo *et al*, 2013) – details are included in the Supplementary Material.

## Results

### Ascertainment of tumour KRAS/BRAF mutation status

In total, 154 patients were enrolled in the study (Köhne *et al*, 2012). One patient withdrew consent and three patients had no DNA and/or tumour tissue available. The *RAS* mutational analysis therefore included 150 patients, of whom 143 received at least one dose of panitumumab.

Overall, 38% of patients had *KRAS* exon 2 mutations, and 10% had *RAS* mutations beyond *KRAS* exon 2 (*KRAS* exon 3 and 4 mutations each in 3% of tumours; *NRAS* exon 2 and 3 mutations each in 2% of tumours; no tumour was found to carry an *NRAS* exon 4 mutation). Complete *RAS* data were available for 143 of 150 patients, of whom 69 (45%) had *RAS* WT tumours (i.e., WT for exons 2, 3 and 4 of both *KRAS* and *NRAS*). *BRAF* mutations were present in nine patients (6%), all of whom had tumours that were WT for *RAS*.

### Patients

Baseline characteristics were generally well balanced between patients with *RAS* WT *vs RAS* MT mCRC, and between patients with *RAS* WT/*BRAF* WT and *RAS* or *BRAF* MT status (Table 1). More patients with *RAS* WT and *RAS* WT/*BRAF* WT mCRC had liver-limited metastases, whereas more *RAS* MT and *RAS* or *BRAF* MT patients had metastases at other sites only. The sum of the longest diameters of measurable target lesions was also slightly larger in patients with *RAS* WT and *RAS* WT/*BRAF* WT mCRC compared with the corresponding MT populations. The median follow-up time in the study was 34.0 weeks (range, 5–223 weeks).

### Efficacy

Overall, 141 patients were evaluable for response assessment. Objective responses occurred more frequently in patients with *RAS* WT (59%) *vs RAS* MT (41%) mCRC, and in patients with *RAS* WT/*BRAF* WT (68%) *vs RAS* or *BRAF* MT (37%) tumours (Table 2). Disease control rates were similar between patients with *RAS* WT (91%) and MT (92%) mCRC and were slightly higher in the *RAS* WT/*BRAF* WT (95%) group than in those with *RAS* or *BRAF* MT (89%) mCRC (Table 2).

**Figure 1:**
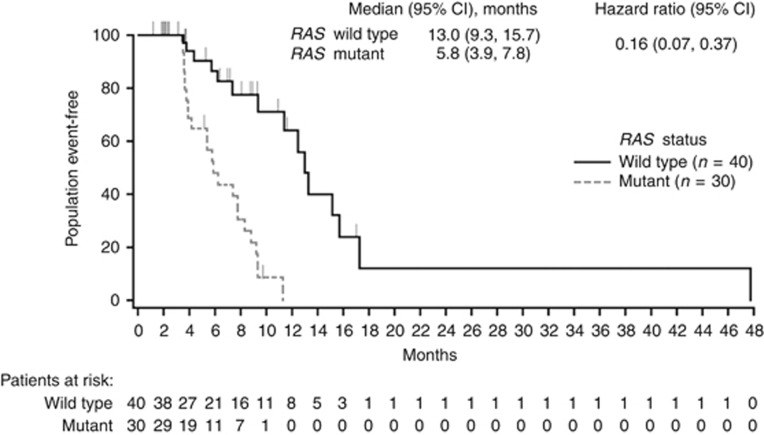
Duration of response by tumour *RAS* status.

The DoR analysis included only those patients who experienced a partial or complete response: 40 patients with mCRC WT for both *RAS* and *BRAF*, and 30 patients with MT *RAS* and WT *BRAF* mCRC (no patient with *BRAF* MT disease responded to treatment). Median DoR was longer in *RAS* WT (13.0 months) *vs RAS* MT (5.8 months) disease (HR, 0.16 (95% confidence interval (CI): 0.07, 0.37)) (Figure 1).

Median DpR (*n*=141) was also significantly greater in patients with *RAS* WT (59.3% (Q1, Q3: 26.4, 77.0%)) than in those with *RAS* MT (35.7% (18.8, 62.0%); *P*=0.0181) disease. Early tumour shrinkage ⩾30% was reported in 49% of patients with *RAS* WT disease and 37% of those with *RAS* MT disease (odds ratio, 1.6), while ETS ⩾20% was reported in 74 and 50% of patients, respectively (odds ratio: 2.8). Early tumour shrinkage was associated with longer PFS in the *RAS* WT *vs* MT groups (⩾30% cutoff: median 14.3 *vs* 7.8 months, HR, 0.29; ⩾20% cutoff: 13.3 *vs* 7.3 months, HR, 0.34). Furthermore, when patient groups were combined (*n*=135), patients achieving ETS had significantly longer PFS than those not achieving the relevant ETS criteria (ETS ⩾30% *vs* <30%: median 10.9 *vs* 7.2 months, HR, 0.45, *P*=0.0003; ETS ⩾ 20% *vs* <20%: 9.1 *vs* 6.9 months, HR, 0.48, *P*=0.0005).

Metastasis resection rates (*n*=143) were numerically higher in patients with *RAS* WT (*n*=9; 13% (95% CI: 6.1, 23.3)) *vs RAS* MT mCRC (*n*=7; 9% (3.9, 18.5)) and were also higher in patients with *RAS* WT/*BRAF* WT (*n*=9; 15% (7.1, 26.6)) *vs* those with *RAS* or *BRAF* MT status (*n*=7; 8% (3.5, 16.6)). Rates of complete resection were similar across the four groups (*RAS* WT, *n*=4, 6% (95% CI: 1.6, 14.2); *RAS* MT, *n*=5, 7% (2.2, 15.1); *RAS* WT*/BRAF* WT, *n*=4, 7% (1.9, 16.2); *RAS* or *BRAF* MT, *n*=5, 6% (2.0, 13.5)).

**Figure 2:**
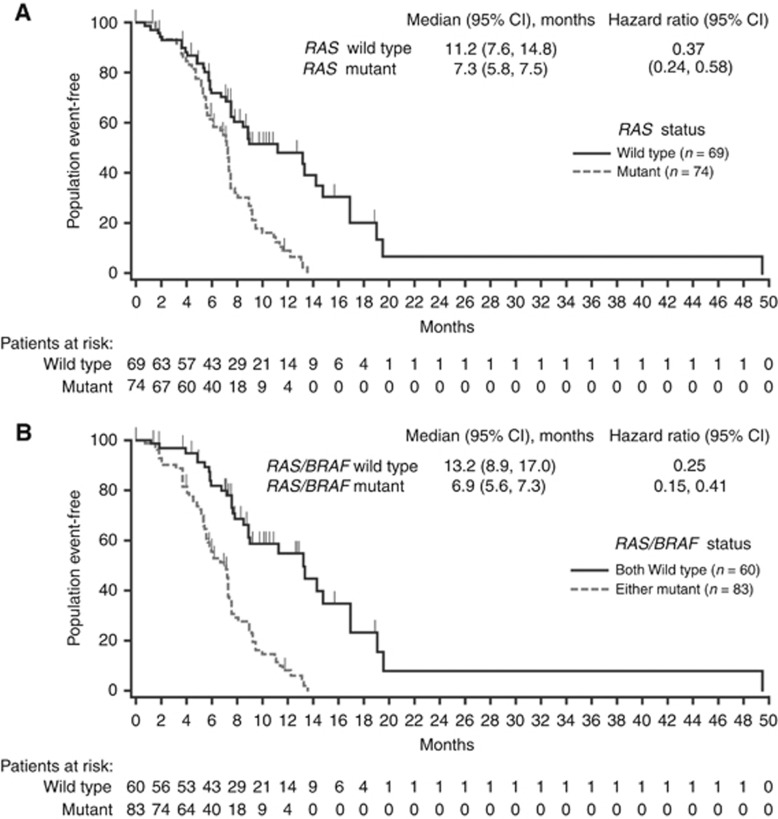
Progression-free survival by tumour (**A**) *RAS* and (**B**) *RAS/BRAF* status.

Median PFS (*n*=143) was longer in patients with *RAS* WT *vs* MT status (11.2 *vs* 7.3 months, respectively; HR, 0.37 (95% CI: 0.24, 0.58)) (Figure 2A) and was also longer in patients with *RAS* WT/*BRAF* WT *vs RAS* or *BRAF* MT mCRC (13.2 *vs* 6.9 months, respectively; HR, 0.25 (95% CI: 0.15, 0.41)) (Figure 2B). Median TTP (*n*=143) was longer in patients with *RAS* WT (13.2 (95% CI: 7.8, 17.0) months) *vs* MT tumours (7.3 (95% CI: 6.1, 7.6) months) and in those with *RAS* WT/*BRAF* WT (13.3 (95% CI: 9.0, 17.0) months) *vs RAS* or *BRAF* MT tumours (7.2 (95% CI: 5.7, 7.4) months).

Median overall survival (*n*=143) was not reached in any of the four *RAS*/*BRAF* groups, although the number of deaths was numerically lower in the *RAS* WT (*n*=5; 7%) and *RAS* WT*/BRAF* WT (*n*=4; 7%) groups *vs* the *RAS* MT (*n*=11; 15%) and *RAS* or *BRAF* MT (*n*=12; 14%) groups (Cox proportional HR, *RAS* WT *vs RAS* MT=0.42 (95% CI: 0.15, 1.23); *RAS* WT*/BRAF* WT *vs RAS* or *BRAF* MT=0.37 (95% CI: 0.12, 1.15)).

### Safety

For patients with *RAS* WT tumours (*n*=69), the median (range) cumulative dose of panitumumab delivered (adjusted for weight) was 72.0 (6.0–395.5) mg kg^−1^. The median (range) relative dose intensity (RDI) was 89% (35–101). For patients with *RAS* MT tumours (*n*=74), the median (range) adjusted cumulative panitumumab dose was 60.4 (5.8–150.0) mg kg^−1^, with a median (range) RDI of 90% (45–103). In the *RAS* WT*/BRAF* WT and MT groups, the median (range) adjusted cumulative panitumumab dose was 74.8 (6.0, 395.5) and 58.0 (5.8, 150.0) mg kg^−1^, respectively, while the median (range) RDI was 89% (35–101) and 90% (45–103), respectively.

The incidence of AEs was similar regardless of tumour *RAS/BRAF* status (Table 3), and no new safety signals were noted in these analyses. AEs leading to study discontinuation occurred in 24–32% of patients across *RAS/BRAF* groups (Table 3), and treatment-related AEs leading to study discontinuation occurred in 16–27% of patients across *RAS/BRAF* groups (Table 3).

Skin toxicity of any grade occurred in 68 of 69 patients (99%) with *RAS* WT tumours, 71 of 74 patients (96%) with *RAS* MT tumours, 59 of 60 patients (98%) with *RAS* WT/*BRAF* WT tumours and 80 of 83 patients (96%) with *RAS* or *BRAF* MT tumours.

### Amphiregulin expression

Of the 154 patients in the study, 100 had evaluable *RAS* status and AREG levels (*RAS* WT, *n*=50; *RAS* MT, *n*=50) (Supplementary Table S1). Tumours with high AREG levels more commonly had WT *RAS* (31 of 50; 62%) while those with low AREG levels more commonly had MT *RAS* (36 of 50; 72%) (Supplementary Table S2). Among *RAS* WT patients, the ORR (95% CI) was 67% (51, 82) in those with high AREG expression and 38% (18, 58) in those with low AREG expression (difference: 29% (2, 53)) (Supplementary Table S3). ORRs in the *RAS* MT group were 31% (10, 53) and 45% (29, 60), respectively (difference, –14% (–39, 15)). The HR for PFS was more favourable in the high AREG group (*RAS* WT/*RAS* MT: 0.30 (95% CI: 0.12, 0.75)) compared with the low AREG group (0.49 (95% CI: 0.21, 1.1)) (Supplementary Figure S1).

The Cox PH model showed a significant *RAS*-by-AREG interaction (*P=*0.03) (Supplementary Table S4). There was a steep transition between responders and non-responders as AREG expression decreased, which was mainly the result of changes in the *RAS* WT group (Supplementary Figure S2).

## Discussion

This analysis represents the first reported *RAS* data beyond *KRAS* exon 2 for panitumumab + FOLFIRI in the first-line treatment of mCRC, an indication that has recently been approved by the EMA. The results show consistently favourable efficacy for first-line panitumumab + FOLFIRI treatment in patients with *RAS* WT/*BRAF* WT tumours compared with MT mCRC tumours. This is consistent with the primary data for the *KRAS* analysis of this study (Köhne *et al*, 2012), in which response rates for patients with *KRAS* WT (*n*=86) and *KRAS* MT (*n*=68) tumours were 56 and 38% and median PFS was 8.9 and 7.2 months, respectively. In the current analysis, extended *RAS* testing identified 69 patients with *RAS* WT tumours, and small increases in response rate (59%) and median PFS (11.2 months) were seen in these patients compared with the primary analysis population. The results are also consistent with previous *RAS* analysis data for panitumumab plus FOLFIRI in the treatment of mCRC (Cohn *et al*, 2011; Mitchell *et al*, 2011; Peeters *et al*, 2014b; Abad *et al*, 2014). Thus, patients whose tumours harbour *RAS* mutations beyond *KRAS* exon 2 are unlikely to benefit from addition of panitumumab to FOLFIRI. Consistent results have also been reported in studies with another anti-EFGR monoclonal antibody, cetuximab (Van Cutsem *et al*, 2015; Heinemann *et al*, 2014a, b), highlighting the importance of up-front tumour *RAS* testing in patients being considered for EGFR inhibitor therapy. As OS was neither an end point nor followed in this trial, efficacy of panitumumab + FOLFIRI was consistent with that reported for *RAS* WT populations in first-line studies of panitumumab + FOLFOX4 (Douillard *et al*, 2013; Schwartzberg *et al*, 2014; Douillard *et al*, 2015), as well as in studies of cetuximab in combination with FOLFIRI or FOLFOX4 (Van Cutsem *et al*, 2015; Bokemeyer *et al*, 2011; Ciardiello *et al*, 2014). For example, in the CRYSTAL study of FOLFIRI plus cetuximab, the PFS in patients with WT *RAS* (11.4 months) and MT *RAS* (7.4 months) (Van Cutsem *et al*, 2015) was almost identical to that in the present study (11.2 and 7.3 months, respectively). The results of the present study are also consistent with previous data showing that *BRAF* mutations are associated with poor prognosis in mCRC regardless of first-line treatment (Phipps *et al*, 2012; Morris *et al*, 2014). Discussion is ongoing, however, regarding the potential usefulness of EGFR inhibitors in patients with *RAS* WT/*BRAF* MT mCRC, Two recent meta-analyses, have reached differing conclusions regarding the predictive role of *BRAF* mutations in patients receiving EGFR inhibitor therapy. While Pietrantonio *et al* (2015) focus on the lack of significant ORR, PFS or OS benefits on addition of EGFR inhibitors to chemotherapy, Rowland *et al* (2015), who included trials of first- and second-line treatment, conclude that there is insufficient evidence to demonstrate a different treatment benefit between patients with *RAS* WT/*BRAF* WT and *RAS* WT/*BRAF* MT disease, and therefore insufficient data to exclude patients with *RAS* WT/*BRAF* MT from EGFR inhibitor therapy. Regardless, such patients should therefore be considered to be at high risk of rapid progression and should be managed accordingly.

No new safety signals were seen with the combination of panitumumab + FOLFIRI in *RAS* WT/*BRAF* WT population; AEs were similar to those seen in the *KRAS* exon 2 WT population of this study (Thaler *et al*, 2012) and in previous studies using this combination in patients with mCRC (Cohn *et al*, 2011; Mitchell *et al*, 2011; Peeters *et al*, 2014b). Overall 28% of patients withdrew from study treatment because of AEs. Consistent with existing data on EGFR inhibitors, there was a high incidence of skin toxicity. While there was no protocol-mandated proactive management of skin toxicity in the present study, it is now recommended for patients receiving EGFR inhibitors (Boone *et al*, 2007; Melosky *et al*, 2009). An earlier analysis of data from the present study showed a higher incidence of skin toxicity in patients with *KRAS* WT tumours than in those with *KRAS* MT tumours (Thaler *et al*, 2012), which may reflect the higher mean cumulative panitumumab dose and longer duration of treatment (i.e., panitumumab cycles) received by the *KRAS* WT group. However, exposure-adjusted AE rates showed integument-related toxicity overall to be higher in the *KRAS* MT *vs* WT population. Thus there is no evidence of an association between tumour *KRAS/RAS* status and toxicity. Furthermore, despite the high incidence of skin toxicity, generic quality of life (QoL) instruments have shown no impact of EGFR inhibitors plus FOLFIRI on overall QoL (Melosky *et al*, 2009; Bennett *et al*, 2011; Thaler *et al*, 2012). Although proactive management of skin toxicity may have reduced the impact for patients, it may also be that the QoL tools used in the study provided too general an assessment to determine the true impact of this AE. Future trials of EGFR inhibitors should include skin-toxicity specific QoL assessment tools to support better understanding of the true impact of this AE on patient wellbeing.

Among patients with *RAS* WT mCRC, high AREG expression was associated with response to panitumumab + FOLFIRI. Consistent with other studies (Jacobs *et al*, 2009; Baker *et al*, 2011; Pentheroudakis *et al*, 2013; Loupakis *et al*, 2014; Jonker *et al*, 2014; Stahler *et al*, 2016), there was an interaction between *RAS* and AREG levels. A higher percentage of patients with *RAS* WT mCRC had high AREG levels compared with those with *RAS* MT mCRC, suggesting that AREG levels are associated with EGFR signalling. Further studies are needed to determine whether there is an AREG expression level below which there is little or no response to panitumumab + FOLFIRI treatment. Biomarker studies remain critical to our understanding of targeted agents in mCRC, and warrant further investigation.

One of the strengths of this comprehensive study was the high percentage of patients from the original cohort who were available for *RAS/BRAF* testing – which was conducted centrally for all specimens – allowing the identification of additional risks for lack of response to treatment. It should be noted that the analyses were retrospective and exploratory in nature, although, as noted above, the results were consistent with previous analyses of efficacy by *RAS* mutation status in patients with mCRC.

In conclusion, first-line panitumumab + FOLFIRI was associated with consistently favourable efficacy in patients with *RAS* WT/*BRAF* WT *vs* MT mCRC tumours and was well tolerated, despite the expected high incidence of skin toxicity. The combination of first-line panitumumab + FOLFIRI also gave efficacy similar to that seen in the *RAS* WT populations in other first-line studies of EGFR-targeted agents plus FOLFIRI or FOLFOX. As per the licensed indication for panitumumab, therefore, patients with *RAS* mutations should not receive panitumumab treatment. Across all lines of therapy, determination of tumour *RAS* status improves identification of patients unlikely to respond to treatment with panitumumab compared with evaluation of *KRAS* exon 2 alone. The combination of panitumumab with FOLFIRI can be considered as an important treatment option for first-line patients with *RAS* WT*/BRAF* WT mCRC.

## Figures and Tables

**Table 1:** Baseline demographics and disease characteristics

	**Panitumumab+FOLFIRI (*****n*****=143)**			
	***RAS*** **WT (*****n*****=69)**	***RAS*** **MT (*****n*****=74)**	***RAS*** **WT/*****BRAF*** **WT (*****n*****=60)**	***RAS*** **or** ***BRAF*** **MT (*****n*****=83)**
Male sex, *n* (%)	55 (80)	42 (57)	50 (83)	47 (57)
White ethnicity, *n* (%)	66 (96)	73 (99)	57 (95)	82 (99)
Age (years), median (range)	62.2 (38–84)	63.7 (37–80)	64.5 (38–84)	64.0 (37–80)
ECOG PS, *n* (%)				
0/1	66 (96)	69 (93)	57 (95)	78 (94)
2	3 (4)	5 (7)^a^	3 (5)	5 (6)^a^
Primary tumour, *n* (%)				
Colon	40 (58)	48 (65)	34 (57)	54 (65)
Rectum	29 (42)	26 (35)	26 (43)	29 (35)
Time since mCRC diagnosis (months),^b^ median (range)	1.1 (0–29)	1.2 (0–44)	1.0 (0–29)	1.2 (0–44)
Number of metastatic sites, *n* (%)				
1	30 (43)	31 (42)	27 (45)	34 (41)
2	22 (32)	25 (34)	18 (30)	29 (35)
⩾3	17 (25)	18 (24)	15 (25)	20 (24)
Sites of metastases, *n* (%)				
Liver only	26 (38)	20 (27)	24 (40)	22 (27)
Liver + other	33 (48)	35 (47)	29 (48)	39 (47)
Other only	10 (14)	19 (26)	7 (12)	22 (27)
Sum of longest diameters of measurable lesions (mm),^c^ median (range)	135.5 (20–381)	107.0 (20–371)	136.0 (20–381)	104.0 (20–371)

Abbreviations: ECOG PS=Eastern Cooperative Oncology Group performance status; mCRC=metastatic colorectal cancer; MT=mutant; WT=wild type.

a One patient in the *RAS* mutant group had an ECOG PS of 3.

b Date of enrolment minus date of primary diagnosis or metastatic disease.

c Target lesions only.

**Table 2:** Best response, objective response and disease control rates

	**Panitumumab + FOLFIRI (*****n***=**141)**			
	***RAS*** **WT (*****n*****=68)**	***RAS*** **MT (*****n*****=73)**	***RAS*** **WT/*****RAF*** **WT (*****n*****=59)**	***RAS*** **or** ***BRAF*** **MT (*****n*****=82)**
Best response, *n* (%)				
Complete response	2 (3)	1 (1)	2 (3)	1 (1)
Partial response	38 (56)	29 (40)	38 (64)	29 (35)
Stable disease	22 (32)	37 (51)	16 (27)	43 (52)
Disease progression	5 (7)	3 (4)	2 (3)	6 (7)
Unevaluable/not done	1 (1)	3 (4)	1 (2)	3 (4)
**Objective response, *n* (%) [95% CI]**	40 (59) [46.2, 70.6]	30 (41) [29.7, 53.2]	40 (68) [54.4, 79.4]	30 (37) [26.2, 48.0]
**Unadjusted odds ratio (95% CI)**	2.0 (1.0, 4.2)		3.7 (1.7, 7.9)	
**Disease control, *n* (%) [95% CI]**	62 (91) [81.8, 96.7]	67 (92) [83.0, 96.9]	56 (95) [85.9, 98.9]	73 (89) [80.2, 94.9]
**Unadjusted odds ratio (95% CI)**	0.9 (0.2, 3.7)		2.3 (0.5, 13.8)	

Abbreviations: CI=confidence intervals; MT=mutant; WT=wild type.

**Table 3:** Summary of adverse events

	**Panitumumab + FOLFIRI (*****n***=**143)**			
	***RAS*** **WT (*****n*****=69)**	***RAS*** **MT (*****n*****=74)**	***RAS*** **WT/*****RAF*** **WT (*****n*****=60)**	***RAS*** **or** ***BRAF*** **MT (*****n*****=83)**
**Any AE,** ***n*** **(%)**	69 (100)	74 (100)	60 (100)	83 (100)
Worst grade ⩾ 3	59 (86)	57 (77)	52 (87)	64 (77)
Serious AE	40 (58)	38 (51)	32 (53)	46 (55)
AEs leading to discontinuation^a^	21 (30)	18 (24)	19 (32)	20 (24)
**Any treatment-related AE, *n* (%)**	69 (100)	74 (100)	60 (100)	83 (100)
Worst grade ⩾3	50 (72)	50 (68)	44 (73)	56 (67)
Serious AE	20 (29)	18 (24)	14 (23)	24 (29)
AEs leading to discontinuation^a^	16 (23)	13 (18)	16 (27)	13 (16)

Abbreviations: AE=adverse event; MT=mutant; WT=wild type.

a Permanent discontinuation of any study drug.
