# Supplementary material for: Impact of tumour RAS/BRAF status in a first-line study of panitumumab + FOLFIRI in patients with metastatic colorectal cancer
Source: Br J Cancer. 2016 Oct 20;115(10):1215–22. doi: 10.1038/bjc.2016.343 (PMC5104899; doi:10.1038/bjc.2016.343)
Supplement: Supplementary Information [file bjc2016343x1.doc]

### SUPPLEMENTARY APPENDIX

**Amphiregulin analysis – methods.** Formalin-fixed paraffin-embedded (FFPE) tissue samples were macrodissected to enrich for tumour content, with non-tumour tissue removed from each slide by a pathologist. Total RNA was then extracted using the High Pure RNA Paraffin Kit (Roche) and quantified by taking optical density (OD260) readings. Complementary deoxyribonucleic acid (DNA) was synthesised from the RNA using the High-Capacity cDNA Archive Kit (Life Technologies). Qualified reverse transcription quantitative polymerase chain reaction (RT-qPCR) assays were used to measure AREG RNA expression. Amphiregulin (AREG) levels were categorised as high or low based on the level derived from an earlier study [Lacouture *et al*., 2010] that best identified treatment responders. Using the *RAS* mutant subgroup as a non-responding comparator, Cox proportional hazards (PH) models were used to evaluate AREG expression levels as a continuous covariate. Decision curves were used to estimate the progression-free survival (PFS) hazard ratio (HR) with increasing levels of baseline AREG expression.

In the Gaussian Process (GP) model the log-linear model is replaced with a compound covariance function using the GPstuff toolkit for MATLAB [Vanhatalo *et al*., 2013]. The baseline hazard part of the model has separate additive covariance functions for a constant, a linear and a non-linear Matern Terms, and the proportional part of the model has a squared exponential covariance function. These were selected based on an extensive search for a combination of covariance functions that maximized the mean predictive density [Vehtari and Lampinen, 2002]. The advantage of the GP model compared with a standard model is that the GP model is more flexible and allows for saturating effects of the biomarker. Thus the curve is not assumed to follow a log-linear relationship as it is in a standard model.

Response rates were estimated using a Bayesian Binomial with a uniform Beta prior. The 95% confidence intervals are calculated where the end-points have equal densities. The differences and the associated intervals are estimated by simulating data drawn from the posterior distributions of each biomarker group.

#### **REFERENCES**

Lacouture ME, Mitchell EP, Piperdi B, Pillai MV, Shearer H, Iannotti N, Xu F, Yassine M (2010) Skin toxicity evaluation protocol with panitumumab (STEPP), a phase II, open-label, randomized trial evaluating the impact of a pre-Emptive Skin treatment regimen on skin toxicities and quality of life in patients with metastatic colorectal cancer. *J Clin Oncol* **28**: 1351 - 1357, doi: 10.1200/JCO.2008.21.7828

Vanhatalo J, Riihimäki J, Hartikainen J, Jylänki P, Tolvanen V, Vehtari A (2013) GPstuff: bayesian modeling with Gaussian processes. *J Mach Learn Res* **14**: 1179.

Vehtari A, Lampinen J (2002) Bayesian model assessment and comparison using cross-validation predictive densities. *Neural Comput* **14**: 2439 - 2468

### Figure legends

**Supplementary Figure 1**. Kaplan–Meier plots of progression-free survival in patients with (**A**) high, (**B**) low, and (**C**) unevaluable amphiregulin levels, categorised by tumour *RAS* status

**Supplementary Figure 2**. Predicted (**A**) relative hazard for patients with *RAS* wild-type and mutant tumours, and (**B**) hazard ratio (wild type/mutant), as functions of AREG expression (decreasing to the right) based on the fitted Gaussian process model. The open circles

represent observed AREG expression values

**Supplementary Table 1**. **Baseline demographics by tumour amphiregulin and *RAS* status.**

|  | ***RAS* wild type** | | | ***RAS* mutant** | | |
| --- | --- | --- | --- | --- | --- | --- |
|  | **Low AREG** | **High AREG** | **Unevaluable AREG** | **Low AREG** | **High AREG** | **Unevaluable AREG** |
| Male sex, *n* (%) | 16 (84) | 25 (81) | 14 (73) | 20 (56) | 8 (57) | 24 (58) |
| White ethnicity, *n* (%) | 18 (95) | 39 (97) | 18 (95) | 36 (100) | 14 (100) | 23 (96) |
| Age (years), median (range) | 62 (38–84) | 65 (47–78) | 66 (38–73) | 64 (44–79) | 58 (48–76) | 70 (37–80) |
| ECOG PS, *n* (%)  0/1  2 | 18 (95)  1 (5) | 29 (93)  2 (7) | 19 (100)  0 | 35 (97)  1 (3) | 13 (93)  1 (7) | 21 (87)  3 (13) |
| Primary tumour, *n* (%)  Colon  Rectum | 13 (68)  6 (32) | 18 (58)  13 (42) | 9 (47)  10 (53) | 25 (69)  11 (31) | 8 (57)  6 (43) | 15 (63)  9 (37) |

Abbreviations: AREG = amphiregulin; ECOG PS = Eastern Cooperative Oncology Group performance status.

**Supplementary Table 2**. **Distribution of amphiregulin levels by *RAS* status.**

|  | ***RAS* wild type** | ***RAS* mutant** |
| --- | --- | --- |
| **AREG biomarker set**  Low AREG^a^  High AREG | **50**  19  31 | **50**  36  14 |
| **Excluded from biomarker set**  Insufficient RNA  Sample unavailable | **19**  4  15 | **24**  5  19 |
| **Total (*n* = 154)** | **69** | **74** |

^a^Based on predetermined cut-off points (ΔCT < 2.09).

Abbreviations: AREG = amphiregulin; ΔCT = change in threshold cycle.

**Supplementary Table 3. Objective response in patients categorised by tumour *RAS* and amphiregulin status.**

|  | ***RAS* wild type** | | | ***RAS* mutant** | | |
| --- | --- | --- | --- | --- | --- | --- |
|  | **Low AREG (*N* = 19)** | **High AREG (*N* = 31)** | **Unevaluable AREG (*N* = 19)** | **Low AREG**  **(*N* = 36)** | **High AREG (*N* = 14)** | **Unevaluable AREG (*N* = 24)** |
| Patients responding | 7 | 21 | 13 | 16 | 4 | 10 |
| Rate, % (95% CI) | 38 (18, 58) | 67 (51, 82) | 67 (47, 86) | 45 (29, 60) | 31 (10, 53) | 42 (24, 61) |
| Difference (high *vs* low) | 29 (2, 53) | |  | –13 (–39, 15) | |  |

Abbreviations: AREG = amphiregulin; CI = confidence interval.

**Supplementary Table 4**. **Interaction testing: *RAS*/AREG (Cox proportional hazards model)a.**

|  | **Estimate (SE)** | ***P*-value** |
| --- | --- | --- |
| AREG | 0.07 (0.11) | 0.55 |
| *RAS* status | 0.47 (0.15) | < 0.01 |
| AREG × *RAS* status | –0.25 (0.12) | 0.03 |

^a^Cox model contains terms for biomarker as a continuous variable, *RAS* status, and the interaction between biomarker and *RAS*. *RAS* wild type was coded as 1, and *RAS* mutant was coded as –1.

Abbreviations: AREG = amphiregulin; SE = standard error.
