# Supplementary figures and images for: Impact of tumour RAS/BRAF status in a first-line study of panitumumab + FOLFIRI in patients with metastatic colorectal cancer
Source: Br J Cancer. 2016 Oct 20;115(10):1215–22. doi: 10.1038/bjc.2016.343 (PMC5104899; doi:10.1038/bjc.2016.343)

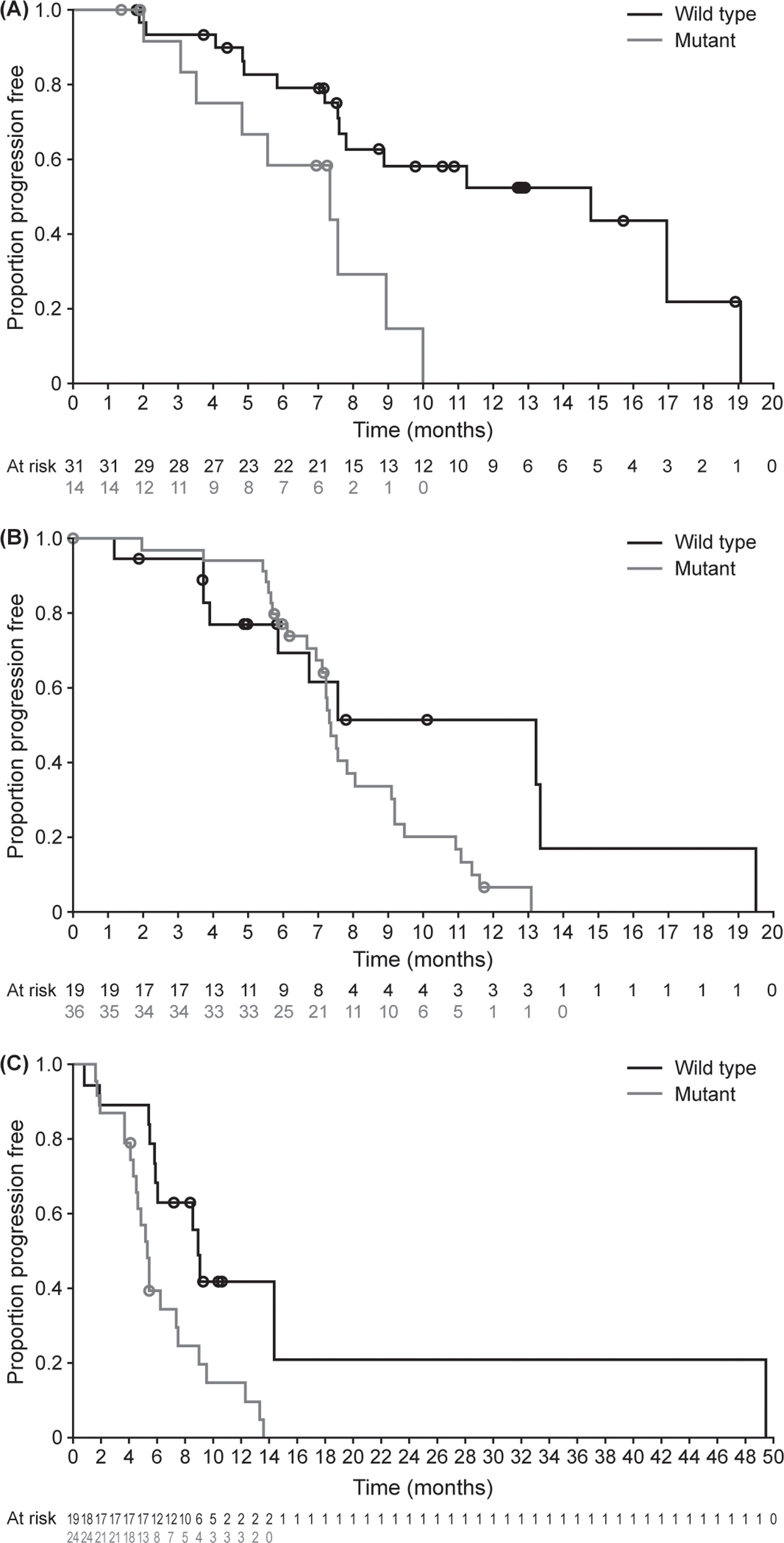

Supplement: Supplementary Figure 1 [file bjc2016343x2.tif]

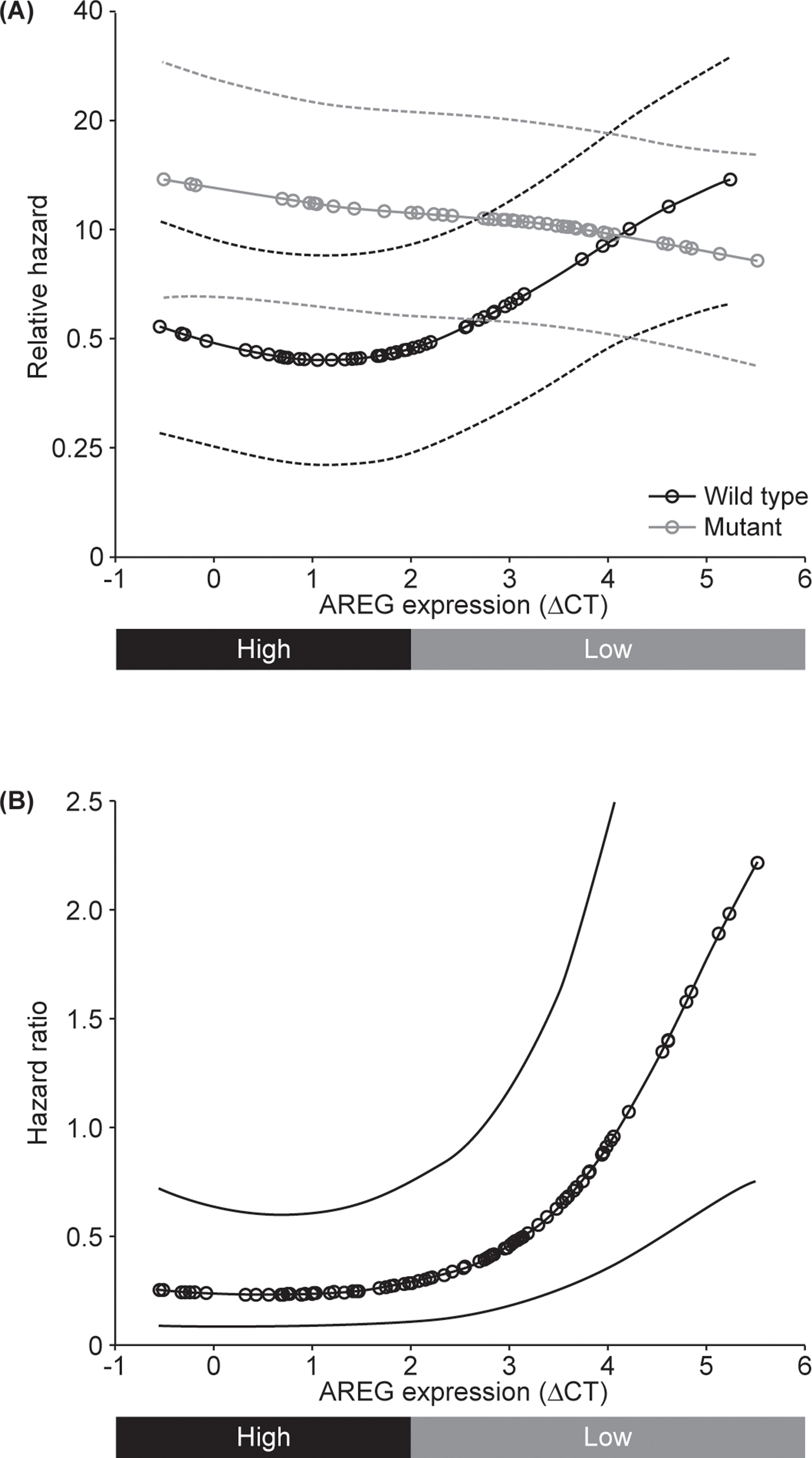

Supplement: Supplementary Figure 2 [file bjc2016343x3.tif]
